# Analysis of Flavor Differences Between *Undaria pinnatifida* Produced Using Different Processing Methods and from Different Origins Based on GC-IMS

**DOI:** 10.3390/foods14122107

**Published:** 2025-06-16

**Authors:** Xinyi Che, Fangjie Cao, Tingmei Yan, Xingyu Liu, Qiming Cai, Shu Liu, Yichao Ma, Dandan Ren, Hui Zhou, Qiukuan Wang, Yunhai He, Han Zhang

**Affiliations:** 1 College of Food Science and Engineering, Dalian Ocean University, Dalian 116023, China; 18141127650@163.com (X.C.); cfj18226792740@163.com (F.C.); m15524912507@163.com (T.Y.); lxy_6826@163.com (X.L.); cqmnbnbznb@163.com (Q.C.); liushu@dlou.edu.cn (S.L.); 18640876812@163.com (Y.M.); rdd@dlou.edu.cn (D.R.); zhouhui@dlou.edu.cn (H.Z.); wqk320@dlou.edu.cn (Q.W.); 2 Key Laboratory of Aquatic Product Processing and Utilization of Liaoning Province, Dalian Ocean University, Dalian 116023, China; 3 National R&D Branch Center for Seaweed Processing, Dalian Ocean University, Dalian 116023, China

**Keywords:** *U. pinnatifida*, processing mode, geographical origin, GC-IMS, flavor differences

## Abstract

To investigate the effects of different processing methods on the characteristic flavor of *Undaria pinnatifida*, this study systematically compared the volatile flavor compositions of four sample groups, namely fresh *U. pinnatifida* from Dalian (WD), salted *U. pinnatifida* from Dalian (WY), dried *U. pinnatifida* from Dalian (WG), and fresh *U. pinnatifida* from Shantou (WS), using GC-IMS, combined with relative odor activity value (ROAV) analysis. The results showed that GC-IMS effectively distinguished the volatile profiles of samples subjected to different processing methods, identifying a total of 45 flavor compounds. Aldehydes emerged as the key flavor components, accounting for relative contents of 53.85% (WD), 41.12% (WY), 52.62% (WG), and 45.28% (WS), which were significantly higher than those of other compound classes. The ROAV analysis revealed that 1-octen-3-ol and 1-octen-3-one were the characteristic compounds shared by all four sample groups. Furthermore, distinct processing methods influenced the distribution of saturated aldehydes, esters, and furans, which could serve as key indicators to distinguish between different processing techniques. Multidimensional analysis, including GC-IMS fingerprint visualization and principal component cluster analysis, confirmed significant flavor differences among the samples from different processing methods and origins. This study provides a theoretical basis for the quality control and standardized production of algal-based foods by multidimensionally analyzing flavor evolution in *U. pinnatifida* during processing.

## 1. Introduction

*Undaria pinnatifida* (*U. pinnatifida*), a temperate, large, annual, brown algae, is widely distributed along the western coast of the North Pacific Ocean, particularly in the coastal waters of China, Japan, and Korea [1]. In China, its annual aquaculture production has stabilized at approximately 200,000 tons in recent years [2]. Recognized as an important functional seaweed [3], *U. pinnatifida* is rich in proteins [4], vitamins [5], and essential minerals, such as calcium, iron, and iodine [6]. Moreover, its extracts contain bioactive components, including polyphenols [7], fucoidan [8], and sterols [9], which exhibit antioxidant [10], hypotensive [11], immunomodulatory [12], thyroid function-regulating [13], and antitumor [14] activities.

While much attention has been paid to its nutritional and physiological functions, the sensory qualities, especially aroma and flavor, that critically affect consumer acceptance and product standardization have received less systematic exploration [15,16]. Indeed, the aroma of seaweed products plays a decisive role in shaping consumer perceptions, with previous studies identifying a range of volatile flavor compounds, such as aldehydes, ketones, alcohols, and sulfur-containing compounds, as key contributors to seaweed aroma. For instance, hexanal imparts grassy green notes, 1-octen-3-ol provides mushroom-like odors, and dimethyl sulfide contributes to marine-like sulfur characteristics [17]. These compounds may originate from enzymatic lipid oxidation, microbial activity, or thermally induced reactions [18].

*U. pinnatifida* from Dalian is mainly harvested in the Jinshitan sea area (39°06′ N, 121°54′ E) of the North Yellow Sea, which features a low mean annual water temperature (seasonal range: ~8–20 °C), high latitude, and notable seasonal fluctuations in salinity and nutrient concentrations [19]. To optimize yield and stress tolerance, locally adapted hardy and high-yielding strains are selected, with cultivation employing deep-water longlines or off-bottom hanging nets to minimize the impact of freezing conditions on growth [20]. In contrast, Shantou *U. pinnatifida* grows near Nan’ao Island (23°24′ N, 116°42′ E), where the average annual water temperature ranges from 22 °C to 28 °C, light availability is high, salinity remains stable, and nutrient salt levels are relatively low [21]. Here, culture practices often involve net-box or three-dimensional ecological systems, often involving co-culture with shellfish and fish, along with the selection of heat-resistant, fast-growing southern strains [22]. Furthermore, both regions adapt the cultivation season, culture depth, and sea–land relay modes in response to local temperature and water-quality dynamics, thereby ensuring efficient and stable production.

Fresh *U. pinnatifida* exhibits a mild and pleasant flavor, whereas processed forms, such as salted or dried *U. pinnatifida*, develop stronger and more complex flavor profiles. These differences primarily result from chemical transformations that occur during processing, including Maillard reactions, lipid oxidation, and enzymatic degradation [23,24], all of which significantly alter the composition of volatile compounds. Such reactions promote the formation or breakdown of key aroma-active substances [25], including aldehydes, ketones, and alcohols, thereby shaping the overall flavor characteristics of *U. pinnatifida*.

A variety of techniques exist for analyzing volatile flavor components, each with distinct advantages and limitations. An electronic nose (E-nose) can rapidly screen for overall volatile profiles, but suffers from limited resolution [26]. Gas chromatography–mass spectrometry (GC-MS) provides high sensitivity and resolution, although it requires complex pre-treatment and is relatively costly [27]. Gas chromatography–olfactometry (GC-O) yields intuitive sensory insights into key flavor compounds, but suffers from low throughput [28]. By comparison, headspace gas chromatography–ion mobility spectrometry (GC-IMS) couples GC pre-separation with ion mobility spectrometry, enabling rapid (<20 min/sample), sensitive, and non-destructive analysis of volatile organic compounds (VOCs) at atmospheric pressure with simple pre-treatment, while simultaneously retaining monomer and dimer forms. This technique allows real-time flavor compound profiling and serves as an efficient alternative to traditional GC-MS. GC-IMS has gained in popularity in regard to drug testing, disease monitoring, environmental protection, and, especially, food flavor analysis [29]. However, despite its extensive use for analyzing meat [30] and dairy products [31], GC-IMS application in algal foods remains exploratory.

Therefore, this study employed headspace gas chromatography–ion mobility spectrometry (GC-IMS), combined with relative odor activity value (ROAV) analysis, to systematically compare the volatile flavor profiles of four representative *U. pinnatifida* sample groups: fresh *U. pinnatifida* from Dalian (WD), salted *U. pinnatifida* from Dalian (WY), dried *U. pinnatifida* from Dalian (WG), and fresh *U. pinnatifida* from Shantou (WS). The specific objectives were to: (1) characterize the differences in the volatile compounds among the *U. pinnatifida* samples subjected to different processing methods and from different cultivation regions; and (2) identify and evaluate the major aroma-active compounds contributing to characteristic flavor profiles through ROAV screening. The findings aim to provide a theoretical and analytical basis for the flavor quality control, targeted processing optimization, and regional product standardization of *U. pinnatifida*.

## 2. Materials and Methods

### 2.1. Materials and Reagents

*Undaria pinnatifida* samples were sourced as follows: fresh *U. pinnatifida* from Dalian (WD), salted *U. pinnatifida* from Dalian (WY), dried *U. pinnatifida* from Dalian (WY), and fresh *U. pinnatifida* from Shantou (WS). All the samples were collected on the same day (9 March 2023), temporarily stored at 4 °C, and analyzed within 24 h to ensure consistency and minimize variability. Images of the samples are shown in Figure S1. A FlavourSpec^®^ flavor analyzer (G.A.S., Dortmund, Germany), equipped with a ^3^H radio ionization source and a CTCPAL autosampler (CTC Analytics AG, Zwingen, Switzerland), served as the GC-IMS platform, and demonstrated a limit of detection (LOD) of <1 μg/L, with acetone employed as the calibration standard. An image of the instrument is provided in Figure S2.

### 2.2. Preparation of U. pinnatifida Samples

Referring to the standard used in the aquatic industry in the People’s Republic of China (SC/T 3213-2019) [32], the rehydration conditions for the WG were modified as follows: the sample was soaked in deionized water at a volume 20 times its dry weight (*w*/*v* ratio of 1:14) at room temperature (15–25 °C) for 10 min. After soaking, the WG was blotted to remove excess water and cut into ~1 cm strips. The WD, WS, and WY *U. pinnatifida* were simply sheared into similar strips. All the prepared samples were immediately stored at 4 °C, until GC-IMS measurement.

### 2.3. Analysis Conditions for the GC-IMS

The analysis followed the process described by Zhang et al. [33] with minor modifications. Briefly, 2.0 g of each *U. pinnatifida* sample was placed into a 20 mL headspace vial and equilibrated at 60 °C for 20 min. A heated syringe (85 °C) extracted 500 μL of the headspace gas, which was transferred using high-purity nitrogen (99.99%) into the GC column for separation. The GC column was an MXT-5 column (15 m × 0.53 mm ID × 1.0 μm df; RESTEK, Bellefonte, Pennsylvania, PA, USA). The temperature program was 40 °C (2 min), ramp to 10 mL/min over 8 min, ramp to 100 mL/min over 10 min, and hold at 150 mL/min for 10 min. The IMS inlet temperature was set at 80 °C, the transfer line at 60 °C, and the detector at 45 °C. The data acquisition and processing utilized VOCal0.1.03 software with the Reporter, Gallery Plot, and Dynamic PCA plug-ins.

### 2.4. The Calculation of Relative Odor Activity Values (ROAVs)

The ROAV method quantifies each volatile compound’s contribution to the overall aroma [34]. ROAV_max_ (for the compound with the greatest impact) is set to 100, and all other ROAV_i_ are calculated as follows:

(1) $$\mathrm{ROAV}=\frac{C_{i}}{C_{\max}}\times \frac{T_{\max}}{T_{i}}\times 100$$

where *C*_i_ is the relative content (%) of compound *i*, *T*_i_ is its odor threshold (μg/kg), *C*_max_ is the relative content of the most impactful compound, and *T*_max_ is its threshold. Compounds with an ROAV > 1 are deemed key flavor compounds; those with a 0.1 ≤ ROAV ≤ 1 are considered important.

### 2.5. Statistical Analysis

Each sample was analyzed in triplicate, using the same instrument. Volatile substances were identified using the built-in NIST and IMS libraries. Quantification was performed via standard curves, using LAV’s Reporter plug-in. Two- and three-dimensional GC-IMS spectra and fingerprints were generated with the Gallery Plot plug-in. The Dynamic PCA plug-in was used for the principal component and cluster analyses to visualize and quantify the differences in the VOC profiles among the processing methods.

## 3. Results and Discussion

### 3.1. Spectral Analysis of Volatile Organic Compounds in U. pinnatifida Processed Using Different Methods

**Figure 1:**
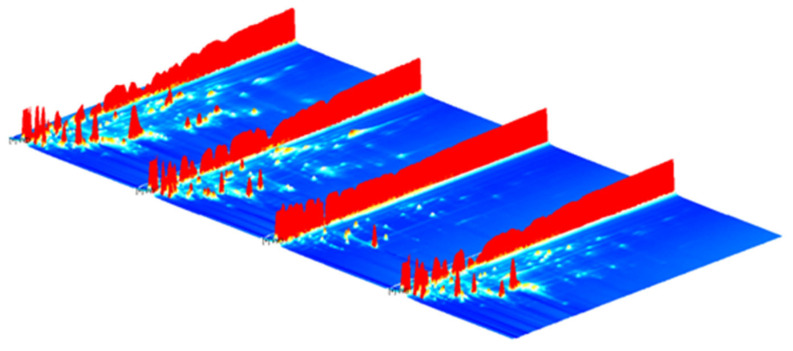
GC-IMS 3D ion mobility spectra of VOCs in WD, WY, WG, and WS samples.

The volatile components of the WD, WY, WG, and WS samples were analyzed using GC-IMS. The three-dimensional ion mobility spectra (Figure 1) were generated by the FlavourSpec^®^ instrument, using the NIST library to match each compound’s drift time, retention index, and migration time [35]. In the resulting maps, the Y-axis represents the retention time (s), the X-axis represents the drift time (ms), and the Z-axis indicates the signal intensity (V). Individual compounds are represented as colored points on a white-to-red scale, where white denotes minimal intensity and red denotes maximum intensity. Notably, each compound may produce multiple signals corresponding to its monomeric, dimeric, or trimeric forms, depending on its concentration in the sample [36]. Figure 1 reveals that the overall signal distributions vary across the processing methods. Samples processed identically display similar spot patterns, indicating comparable volatile profiles and making direct intergroup distinctions difficult. To emphasize the differences, we employed the difference comparison mode to generate two-dimensional top views.

**Figure 2:**
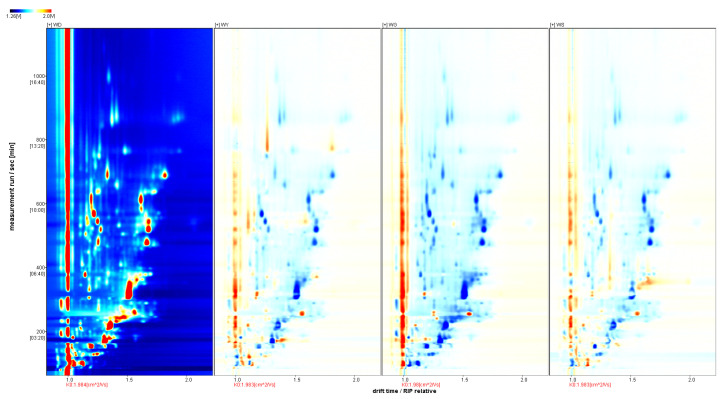
The GC-IMS differential spectra of volatile organic compounds in WD, WY, WG, and WS samples.

In the two-dimensional map (Figure 2), WD serves as the reference sample; the spectra of the WY, WG, and WS samples are subtracted sequentially. The red points of the WY, WG, and WS samples indicate VOCs with higher concentrations than in WD, while the points in blue indicate lower concentrations. The vertical red line at drift = 1.0 corresponds to the reactive ion peak (RIP) after normalization. The color intensity reflects the magnitude of change, with a deeper red signifying a larger increase, and deeper blue signifying a larger decrease relative to the reference sample. As shown in Figure 2, the VOC distributions differ markedly among the four *U. pinnatifida* samples, with the WG sample exhibiting the lowest overall VOC concentration. These results demonstrate that both the processing method and geographic origin play a crucial role in shaping the volatile flavor composition of *U. pinnatifida*, yielding distinct flavor characteristics for each sample group.

### 3.2. Analysis of Volatile Components in Undaria pinnatifida Samples

The volatile flavor compounds in the *U. pinnatifida* samples were analyzed using gas chromatography–ion mobility spectrometry (GC-IMS). Each compound was identified and quantified based on its retention time and ion migration time, with confirmation achieved through the use of the NIST and VOCal IMS databases. The GC-IMS enabled the detection of both simple (monomeric) and more complex (dimeric, trimeric) ion forms. A total of 87 peaks were observed, of which 45 volatile compounds were successfully identified (see Table S1).

These volatiles arise from various metabolic processes during the growth of *U. pinnatifida*. Specifically, they are derived from biochemical transformations of amino acids, fatty acids, sugars, glycosides, and carotenoids. These reactions include deamination, lipid oxidation, and protein breakdown [37]. Notably, the relative concentrations of these compounds differed significantly among the four sample types.

Across all the samples, aldehydes were the most abundant compounds, contributing 41.12–53.85% of the total volatiles. Alcohols (8.24–19.86%) and ketones (3.67–7.88%) followed in terms of abundance. In contrast, esters (0.41–1.02%) and furans (0.41–3.38%) were present in much smaller amounts. Due to the sensitivity of GC-IMS, dimeric and trimeric forms were also detected, enabling the improved differentiation of similar compounds, an advantage noted in previous food studies [38].

The WD and WG samples had the highest aldehyde content (53.85% and 52.62%, respectively). In contrast, the WY sample had the lowest (41.12%). Notably, (E)-2-hexenal reached 21.13% in the WS sample, possibly due to enhanced lipoxygenase (LOX) and hydroperoxide lyase (HPL) activity under high temperature and light conditions in Shantou [39,40,41]. In the WY sample, salt treatment may have reduced aldehyde formation by inhibiting protease activity and limiting the Strecker degradation pathway [42]. Additionally, salt ions can stabilize lipid peroxides and slow down oxidation [43]. However, (E)-2-pentenal was still detected, likely due to cell membrane damage and continued fatty acid oxidation [24]. The WG sample had the highest level of hexanal (19.05%), significantly more than the WD, WY, or WS samples. Drying likely disrupted the cell membranes and promoted the non-enzymatic oxidation of polyunsaturated fatty acids, producing large amounts of hexanal [44,45,46].

The alcohol content was highest in the WY (19.86%) and WS (18.40%) samples, and lower in the WD (8.24%) and WG (11.28%) samples. The WY sample had elevated ethanol (6.62%), which may stem from microbial fermentation via the pyruvate decarboxylase–ethanol dehydrogenase pathway [47,48,49]. Notably, 1-octen-3-ol, a compound with a mushroom-like aroma, reached 6.47% in the WY sample. It likely formed from linoleic acid via LOX/HPL pathways, activated by salting-induced cell damage [50,51,52]. The WS sample showed 3.05% dimerized 1-hexanol, indicating active LOX-mediated oxidation of fatty acids. The moderate temperature (~22 °C) in the Shantou region may have helped maintain the enzyme activity and promoted dimer formation [53].

The WD and WS samples had the highest ketone contents (7.88% and 6.64%, respectively), mainly due to 1-octen-3-one, derived from fatty acid oxidation. The fresh samples better retained their LOX and HPL activity, while salt or heat treatment suppressed these enzymes [45,54,55]. The WS sample also had unusually high acetone content, possibly from altered sugar metabolism under variable light (via pyruvate pathways) [56], Strecker degradation in ruptured cells [45,57], or UV-induced degradation of β-carotene [57,58].

The WG and WS samples had the highest ester levels, especially ethyl hexanoate in the WS sample (0.70%). This may relate to enhanced esterase activity under osmotic stress in intertidal environments [59,60,61,62]. The WG sample contained 3.38% 2-pentylfuran, much higher than the other samples. This furan likely formed through Maillard and Strecker reactions during drying, involving degraded sugars and amino acids (e.g., proline, glutamate) [63,64]. Similar mechanisms are seen during tea and pumpkin seed processing.

In order to systematically investigate the flavor changes in *U. pinnatifida*, this study calculated the ROAVs of 45 identified volatile compounds to quantify the contribution of each component to the overall flavor. The odor thresholds for each compound were taken from Odour Thresholds [65]. The ROAV analysis revealed compounds with varying degrees of flavor impact: those with an ROAV > 1 were classified as key flavor compounds, while compounds exhibiting a 0.1 ≤ ROAV ≤ 1 were designated as important flavor contributors [66,67,68]. The ROAVs of the 45 compounds are listed in Table 1.

In this study, three core flavor profile compounds were jointly identified in both the WD and WS processed samples: (E,E)-2,4-nonadienal, imparting a pronounced greasy greenish aroma [69]; 1-octen-3-one, responsible for a typical mushroom earthy aroma [70]; and 3-methylbutyraldehyde, contributing a chocolate-like fatty aroma [71].

Specifically, aldehydes, primarily formed via lipid oxidation, dominated the flavor profile. Notably, (E,E)-2,4-nonadienal showed a significantly higher ROAV in the WS sample than in the WD sample (Table 1), likely due to enhanced linoleic acid oxidation catalyzed by LOX and, subsequent, cleavage by HPL [72,73]. Environmental factors like higher temperatures and salinity may further boost LOX activity and fatty acid degradation [69,74]. (E)-2-nonenal, another key aldehyde, can form through the thermal or photooxidative cleavage of lipid peroxides [75]. Although long-chain aldehydes (e.g., n-nonanal) were abundant, their high odor thresholds limited their sensory impact [76].

Ketones, especially 1-octen-3-one (ROAV = 100), played a major role, due to their extremely low thresholds. They form from linoleic acid via 8-HPODE and subsequent β-cleavage, a pathway favored by the abundance of n-6 polyunsaturated fatty acids in U. pinnatifida [77,78,79,80,81].

Alcohols contributed significantly to the flavor variation between the WS and WD samples. For example, 1-octen-3-ol was more prominent in the WS sample (ROAV = 11.12 vs. 1.74 in the WD sample), likely due to humidity-enhanced LOX/HPL activity [71,81]. Together with 1-octen-3-one, it shaped a mushroom–herb aroma complex [82,83].

Region-specific characteristics were also observed. The WD sample featured a fresher profile with cucumber- and melon-like aromas from (E)-2-nonenal and (E,E)-2,4-hexadienal, reflecting cold water-induced n-3 PUFA autoxidation [41,84,85]. In contrast, the WS sample had a richer aroma from alcohols, ketones, and esters. Notably, ethyl valerate (ROAV = 5.46) and 3-methylbutyraldehyde formed a fruity–chocolatey matrix, likely due to heat-enhanced β-oxidation and AAT-mediated ester biosynthesis [43,45,86,87].

The comparative analysis (Table 1) highlighted five core contributors, namely (E)-2-nonenal, (E,E)-2,4-nonadienal, 1-octen-3-one, 1-octen-3-ol, and ethyl pentanoate, imparting green, greasy, mushroom-like, fruity, and creamy notes, respectively [69].

Drying significantly enhanced aldehyde formation via autoxidation and Maillard pathways. For example, the ROAV of (E,E)-2,4-nonadienal rose from 9.74 (fresh) to 39.98 (salted) and 48.03 (dried) [88,89,90,91,92,93]. Similarly, (E)-2-nonenal increased markedly in the WG sample (ROAV = 43.56), attributed to intensified β-oxidation under low water activity [94,95,96].

Also, 3-methylbutyraldehyde increased during salting and drying, possibly due to the Strecker degradation of valine under high salt and heat conditions [41,97,98,99]. Although the benzaldehyde content rose, its flavor impact remained minor due to it having a high threshold [100].

Among the alcohols, only unsaturated and branched-chain types like 1-octen-3-ol were significant. Its ROAV rose in salted and dried samples, due to LOX activation and enhanced lipid-enzyme interactions during processing [86,101,102,103].

Ketones, such as 1-octen-3-one, were crucial in masking fishy notes. In the WG sample, its ROAV reached 100, linked to hydroperoxide decomposition under thermal dehydration [104,105,106]. Similar increases were observed in the WY sample (ROAV = 51.98), likely due to the salt-induced release of lipid precursors [107,108]. Interestingly, the dimer form of 1-octen-3-one decreased in the WG sample, possibly due to changes in the antioxidant enzyme activity during drying [109].

Esters typically impart pleasant fruity, sweet, and floral notes due to their low odor thresholds. Unsaturated esters generally exhibit lower thresholds than their saturated counterparts [110,111,112]. Esters, while low in overall content [113], gained prominence post-processing. Ethyl valerate showed significant increases in the WG (ROAV = 40.68) and WY (ROAV = 25.52) samples, possibly due to LOX pathway stimulation and enhanced esterification as a result of low water activity and altered membrane permeability [114,115].

Finally, furans like 2-pentylfuran, which contribute roasted and nutty notes, also increased post-drying (ROAV = 3.83 in WG), likely formed via lipid oxidation and Maillard reactions [116,117,118,119].

From a processing perspective, the applied treatments had a profound influence on the flavor profiles of the *U. pinnatifida* samples. Drying, in particular, synergistically enhanced lipid oxidation and Maillard reactions through dehydration and thermal input [120], resulting in the development of a robust composite aroma, characterized by greasy, loamy, and soybean-like notes.

### 3.3. Fingerprint Analysis of Volatile Components in Different Algal Samples

**Figure 3:**
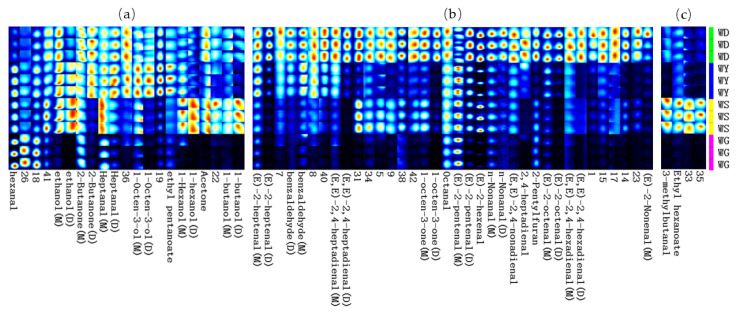
(**a**) Hexanal, ethanol, 2-butanone, heptanal, 1-octen-3-ol, ethyl pentanoate, 1-hexanol, acetone, 1-butanol; (**b**) (E)-2-heptenal, benzaldehyde, (E,E)-2,4-heptadienal, 1-octen-3-one, octanal, (E)-2-pentenal, (E)-2-hexenal, n-nonanal; (E,E)-2,4-nonadienal, 2,4-heptadienal, 2-pentylfuran, (E)-2-octenal, (E,E)-2,4-hexadienal; and (**c**) 3-methylbutanal, ethyl hexanoate. Comparison of volatile organic compound fingerprint spectra for WD, WY, WG, and WS samples.

The GC-IMS fingerprinting revealed distinct differences in the volatile profiles among the four *U. pinnatifida* samples (WD, WY, WG, WS), indicating significant effects of the processing method and regional growing environment on the flavor compound composition. Figure 3 visualizes these differences, with the compound intensity color-coded according to concentration [121].

Figure 3a shows that salting and drying generally increased the concentration of oxidation products, such as hexanal, although the extent varied by sample, likely due to environmental influences on the lipid composition and antioxidant content [9]. Substances like 1-butanol, acetone, and 1-hexanol exhibited differential responses to processing and the environment. For example, the WS sample showed an increase in 1-hexanol, attributed to environmental oxidative stress in the Shantou region [122,123], while drying and salting altered lipid oxidation pathways, reducing small volatiles, due to enzyme inactivation and compound degradation [124,125]. Notably, ethyl valerate and 1-octen-3-ol increased in the salted sample (WY), but decreased in the dried (WG) and Shantou-grown (WS) samples. Salt treatment promoted ester and alcohol production via altered lipid oxidation pathways and membrane permeability [126], while drying likely reduced precursor availability and enzyme activity [127]. Figure 3b shows that several key aldehydes and ketones (e.g., (E,E)-2,4-nonadienal, 1-octen-3-one) decreased significantly across all the processed samples, especially in the WG sample, due to compound volatility and thermal degradation. Conversely, 2-pentylfuran became more prominent post-drying, potentially due to enhanced Maillard reactions [64]. The WS sample showed increases in compounds like ethyl caproate and 3-methylbutyraldehyde, as shown in Figure 3c, likely due to regional microbial activity and proteolysis [128], while the WG sample had elevated glutaraldehyde, contributing to specific flavor notes. The increase in 1-propanol in the WY sample suggests a salting effect, but lacks a flavor impact due to its high sensory threshold.

Overall, the volatile fingerprinting highlights both processing-induced and environment-driven differences in *U. pinnatifida* flavor profiles. Although the fingerprint patterns may differ from the ROAV results due to compound threshold discrepancies, it offers a robust visual means for differentiating between sample profiles.

### 3.4. Cluster Analysis of U. pinnatifida Samples

**Figure 4:**
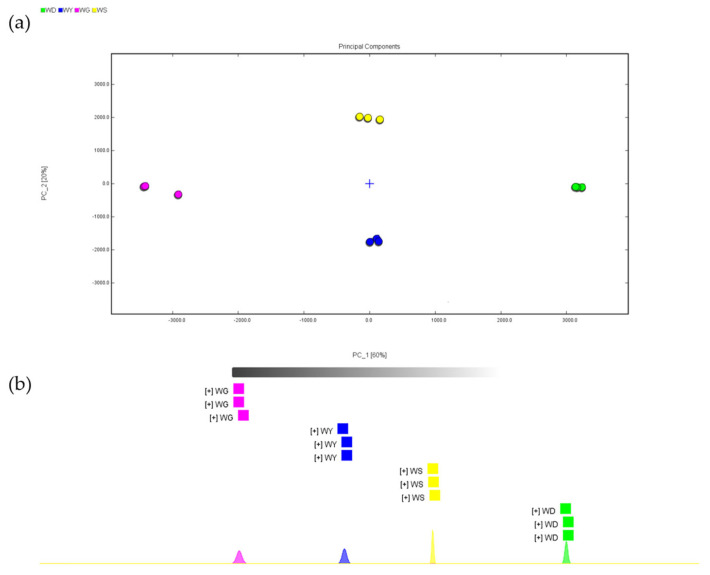
(**a**) Cluster analysis of WD, WY, WG, and WS samples; (**b**) nearest neighbor fingerprint analysis of WD, WY, WG, and WS samples.

To assess the differentiation among the *U. pinnatifida* samples (WD, WY, WG, WS), principal component analysis (PCA) and nearest neighbor fingerprinting were performed, using three replicates per group [129]. As shown in Figure 4, PC1 and PC2 accounted for 60% and 20% of the total variance, respectively, with a cumulative variance of 80%, indicating that the majority of variability in the volatile compound composition could be explained by these two principal components [130,131].

In the PCA score plot (Figure 4a), the WG samples (pink) formed a tightly clustered group, with minimal internal variation, suggesting strong consistency in their volatile profiles. In contrast, the WD (green), WY (blue), and WS (yellow) samples were distinctly separated, with greater internal dispersion, indicating considerable differences in their volatile compositions. The nearest neighbor fingerprinting plot (Figure 4b) corroborated the PCA results, with each sample group forming discrete clusters. This clustering pattern affirms that the processing methods and environmental factors induced substantial divergence in the VOC profiles of *U. pinnatifida*, supporting the differentiation observed in the previous GC-IMS and ROAV analyses.

## 4. Conclusions

In this study, GC-IMS, combined with ROAV analysis, was used to systematically investigate the effects of the processing methods and geographic origin on *U. pinnatifida*’s volatile flavor profile, and to elucidate the formation mechanisms of key aroma compounds. The major findings are summarized as follows:

1. Diverse volatile profiles: Forty-five volatile compounds were identified across all the samples, with aldehydes dominating (41.12–53.85%). Notably, hexanal in the WG sample was six times higher than in the WD sample, while 1-octen-3-ol reached 6.47% in the WY sample. In the WS sample, enhanced lipoxygenase activity led to (E)-2-hexenal comprising 21.47% of the volatiles.

2. Key odorants by the ROAV: (E,E)-2,4-Nonadienal (“greasy” aroma), 1-octen-3-one (“mushroom” aroma), and 3-methylbutyraldehyde (“chocolate” aroma) were identified as core flavor compounds. In the WG sample, 1-octen-3-one achieved the maximum normalized ROAV value of 100, dominating the loamy aroma profile.

3. Processing impacts: Salting reduced certain aldehydes by inhibiting Maillard precursors, but activated microbial pathways to increase ester production (e.g., ethyl valerate’s ROAV rose to 40.68). Drying synergistically enhanced lipid oxidation and Maillard reactions via dehydration and heat, driving an eight-fold increase in 2-pentylfuran and producing a characteristic soybean-like aroma.

4. Regional differences: Shantou’s high-temperature, low-salinity environment favored C6 aldehyde accumulation via the LOX pathway. Dalian’s cold-water conditions promoted C9 aldehyde production, contributing to the fresh, green cucumber notes.

This work establishes a comprehensive volatile flavor database for *U. pinnatifida*, clarifies how processing regulates lipid oxidation and enzymatic pathways, and demonstrates the utility of GC-IMS for algal flavor analysis. However, several limitations should be acknowledged. First, the analysis focused on a limited number of representative samples from two regions, which may not capture the full variability present across broader geographic sources or cultivars. Second, although ROAV provides useful insights into aroma contribution, it does not fully reflect sensory perception, which is also influenced by compound interactions and human variability. Third, environmental and post-harvest factors, such as storage conditions and microbial changes, were not systematically controlled or assessed.

Future research should expand the sample diversity, refine odor-threshold databases to improve ROAV accuracy, and integrate metabolomics for a deeper dissection of flavor-forming pathways, thereby guiding targeted processing innovations and enhancing functional properties.

## Figures and Tables

**Table 1:** ROAVs and total content of various volatile substances.

Classification	Odor Description	Threshold μg/kg	ROAV			
			WD	WY	WG	WS
**Aldehydes**						
(E)-2-Nonenal(M)	fatty, green, waxy, cucumber, melon	0.08	3.94	5.89	8.98	4.27
(E)-2-Nonenal(D)	fatty, green, waxy, cucumber, melon	0.08	1.77	1.68	5.95	1.93
n-Nonanal(M)	rose, citrus, strong oily	1	0.13	0.42	0.89	0.43
n-Nonanal(D)	rose, citrus, strong oily	1	0.03	0.07	0.15	0.06
(E)-2-Octenal(M)	fresh cucumber, fatty, green herbal, banana, green leaf	3	0.12	0.34	0.44	0.18
(E)-2-Octenal(D)	fresh cucumber, fatty, green herbal, banana, green leaf	3	0.12	0.14	0.12	0.04
(E,E)-2,4-Heptadienal(M)	fatty, oily, aldehyde, vegetable, cinnamon	0.01	24.65	93.65	99.86	35.21
(E,E)-2,4-Heptadienal(D)	fatty, oily, aldehyde, vegetable, cinnamon	0.01	37.93	50.72	19.51	10.81
Benzaldehyde(M)	bitter almond, cherry, nutty	350	0.00	0.00	0.00	0.00
Benzaldehyde(D)	bitter almond, cherry, nutty	350	0.00	0.00	0.00	0.00
(E)-2-Heptenal(M)	spicy, green vegetables, fresh, fatty	13	0.02	0.08	0.09	0.04
(E)-2-Heptenal(D)	spicy, green vegetables, fresh, fatty	13	0.04	0.06	0.03	0.02
(E,E)-2,4-Hexadienal(M)	green grassy, fatty, fruity	0.008	7.69	18.69	34.15	15.85
(E,E)-2,4-Hexadienal(D)	green grassy, fatty, fruity	0.008	5.86	4.35	14.42	5.07
(E)-2-Hexenal	green, banana, fat	17	0.15	0.23	0.14	0.59
Hexanal	fresh, green, fat, fruity	4500	0.00	0.00	0.00	0.00
(E)-2-Pentenal(M)	potato, peas	46	0.00	0.02	0.05	0.02
(E)-2-Pentenal(D)	potato, peas	46	0.03	0.07	0.04	0.05
**Aldehydes**						
3-Methylbutanal	chocolate, fat	0.4	0.04	0.05	0.08	0.57
Octanal	aldehyde, waxy, citrus, orange, fruity, fatty	0.7	0.05	0.30	0.80	0.25
2,4-Heptadienal	nut, fat	15.4	0.01	0.02	0.01	0.00
Pentanal	green grassy, faint banana, pungent	20	0.00	0.01	0.06	0.01
Heptanal(M)	fresh, aldehyde, fatty, green herbs, wine, fruity	3	0.02	0.15	0.35	0.16
Heptanal(D)	fresh, aldehyde, fatty, green herbs, wine, fruity	3	0.02	0.17	0.09	0.07
(E,E)-2,4-Nonadienal	fatty, green grassy, cucumber, fishy	0.09	0.90	2.84	2.66	2.59
Total content			53.85%	41.12%	52.62%	45.28%
**Alcohols**						
Ethanol(M)	aromaticity	100,000	0.00	0.00	0.00	0.00
Ethanol(D)	aromaticity	100,000	0.00	0.00	0.00	0.00
1-Octen-3-ol(M)	mushroom, lavender, rose, hay	0.018	10.88	100.00	100.00	69.52
1-Octen-3-ol(D)	mushroom, lavender, rose, hay	0.018	8.87	72.72	15.75	15.65
1-Octen-3-ol(T)	mushroom, lavender, rose, hay	0.018	2.10	9.62	4.56	3.92
1-Hexanol(M)	fresh, fruity, wine, sweet, green	250	0.00	0.00	0.00	0.00
1-Hexanol(D)	fresh, fruity, wine, sweet, green	250	0.00	0.00	0.00	0.01
1-Pentanol(M)	balsamic	4000	0.00	0.00	0.00	0.00
1-Pentanol(D)	balsamic	4000	0.00	0.00	0.00	0.00
1-Butanol(M)	wine	500	0.00	0.00	0.00	0.00
1-Butanol(D)	wine	500	0.00	0.00	0.00	0.00
1-Propanol	alcohol, pungent	53,952.63	0.00	0.00	0.00	0.00
Total content			8.24%	19.86%	11.28%	18.40%
**Ketones**						
1-Octen-3-one(M)	strong earthy, mushroom, vegetable, fishy, chicken	0.005	27.43	39.95	59.84	100.00
1-Octen-3-one(D)	strong earthy, mushroom, vegetable, fishy, chicken	0.005	100.00	76.85	36.19	96.44
2-Butanone(M)	fruity, camphor	1.1	0.07	0.32	0.95	0.41
2-Butanone(D)	fruity, camphor	1.1	0.09	0.53	0.19	0.12
Acetone	fresh, apple, pear	14,500	0.00	0.00	0.00	0.00
Total content			7.88%	5.61%	3.67%	6.64%
**Esters**						
Ethyl hexanoate	fruity, creamy	5	0.01	0.03	0.03	0.07
Ethyl pentanoate	apple, green grassy	1.5	0.02	0.22	0.27	0.06
Total content			0.41%	0.91%	1.02%	0.89%
**Furans**						
2-Pentylfuran	bean, fruity, earthy, green, vegetable	6	0.04	0.03	0.30	0.09
Total content			1.61%	0.41%	3.38%	1.09%

## Data Availability

The original contributions presented in the study are included in the article/Supplementary Material, further inquiries can be directed to the corresponding author.

## Supplementary Materials

The following supporting information can be downloaded at: https://www.mdpi.com/article/10.3390/foods14122107/s1, Figure S1: *Undaria pinnatifida* samples: (**a**) fresh *U. pinnatifida* from Dalian, WD; (**b**) salted *U. pinnatifida* from Dalian, WY; (**c**) dried *U. pinnatifida* from Dalian, WG; (**d**) fresh *U. pinnatifida* from Shantou, WS; Figure S2: GC-IMS instrument; Table S1: Qualitative analysis of WD, WY, WG, and WS VOCs.
