# Supplementary material for: Analysis of Flavor Differences Between Undaria pinnatifida Produced Using Different Processing Methods and from Different Origins Based on GC-IMS
Source: Foods. 2025 Jun 16;14(12):2107. doi: 10.3390/foods14122107 (PMC12192156; doi:10.3390/foods14122107)
Supplement: Supplementary file 1 [file foods-14-02107-s001.zip › foods-3671113-supplementary/Figure S1;Figure S2.pdf]

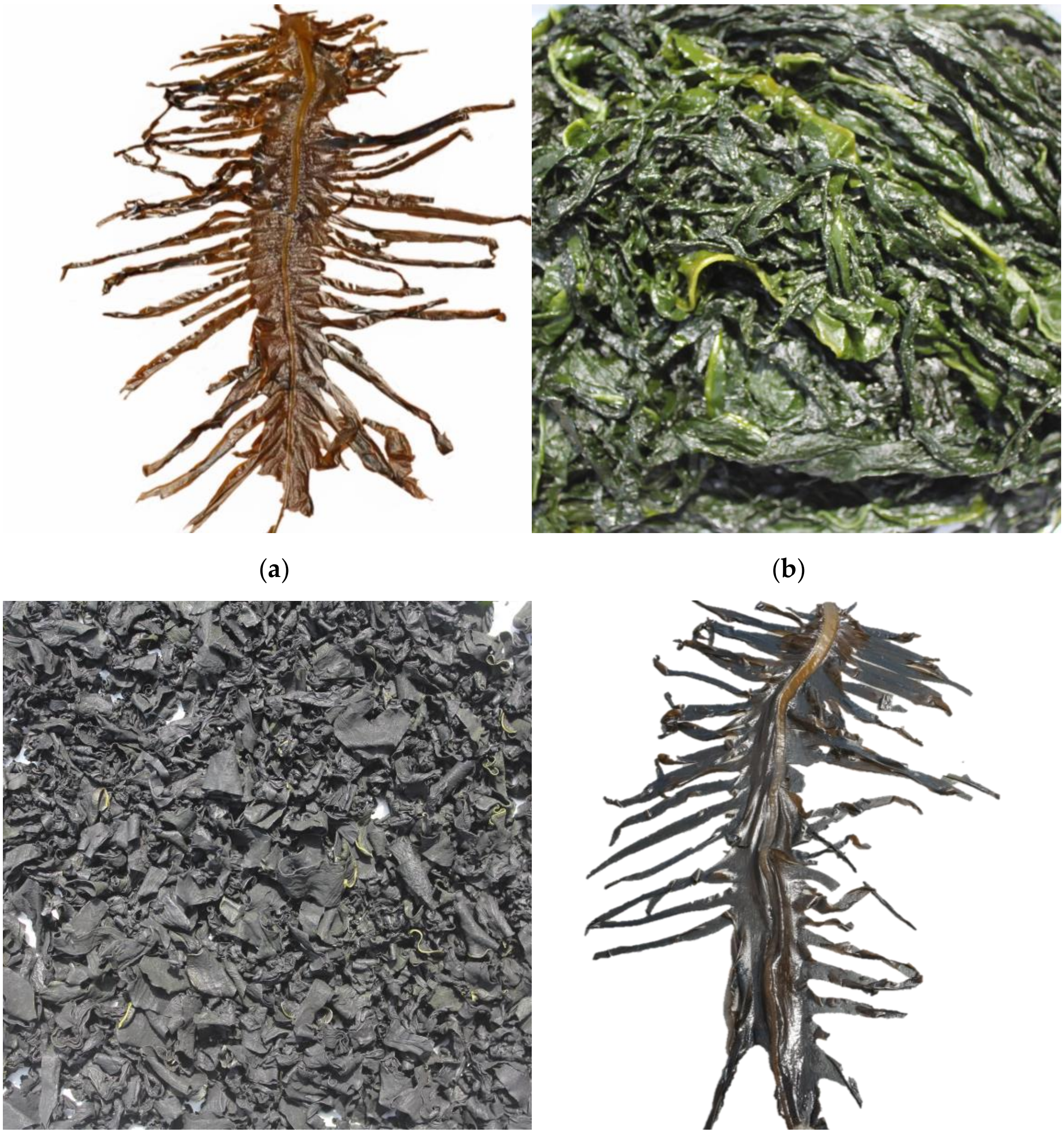

(**a**) (**b**) (**c**) (**d**)

Figure S1. *Undaria pinnatifida* Samples. (**a**) fresh *U. pinnatifida* from Dalian, WD; (**b**) salted *U. pinnatifida* from Dalian, WY; (**c**) dried *U. pinnatifida* from Dalian, WG; (**d**) fresh *U. pinnatifida* from Shantou, WS.

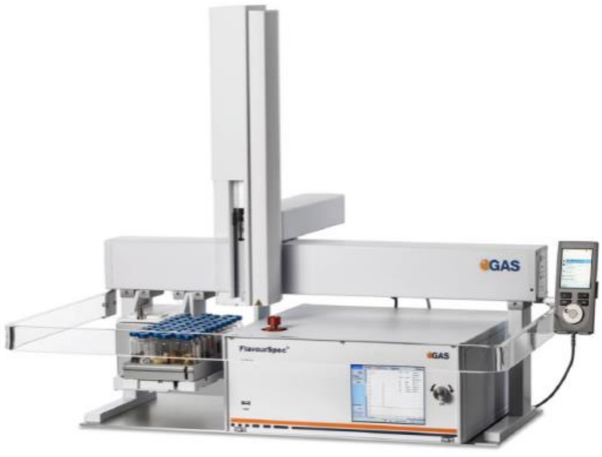

Figure S2. GC-IMS Instrument.
