# Supplementary material for: Analysis of Flavor Differences Between Undaria pinnatifida Produced Using Different Processing Methods and from Different Origins Based on GC-IMS
Source: Foods. 2025 Jun 16;14(12):2107. doi: 10.3390/foods14122107 (PMC12192156; doi:10.3390/foods14122107)
Supplement: Supplementary file 1 [file foods-14-02107-s001.zip › foods-3671113-supplementary/Table S1.pdf]

Table S1. Qualitative analysis of WD, WY, WG and WS VOCs

Volatile compounds	Molecular formula	Retention index	Rt [sec]	Peak Volume				Relative content			
				WD	WY	WG	WS	WD	WY	WG	WS
**Aldehydes**											
(E)-2-Nonenal(M)	C9H16O	1152.4	892.627	3631.74±1478.19a	877.31±130.51b	440.11±36.31b	686.21±14.61b	2.25%	0.93%	1.34%	0.72%
(E)-2-Nonenal(D)	C9H16O	1148.2	881.106	1634.45±1425.23a	250.91±5.01a	291.51±15.61a	310.41±48.71a	1.01%	0.27%	0.89%	0.32%
n-Nonanal(M)	C9H18O	1105.1	770.471	1493.80±34.86a	786.21±46.51b	542.11±13.11c	863.31±47.61b	0.93%	0.83%	1.65%	0.90%
n-Nonanal(D)	C9H18O	1103	765.386	300.83±20.67a	122.21±4.21b	93.21±5.91c	127.51±3.81b	0.19%	0.13%	0.28%	0.13%
(E)-2-Octenal(M)	C8H14O	1070.1	690.955	4023.94±64.55a	1903.71±180.71b	812.91±137.61c	1057.01±51.81c	2.50%	2.01%	2.48%	1.10%
(E)-2-Octenal(D)	C8H14O	1068.1	686.62	4066.33±202.59a	770.31±96.91b	229.61±20.61c	249.51±14.01c	2.52%	0.81%	0.70%	0.26%
(E,E)-2,4-Heptadienal(M)	C7H10O	1031.1	611.946	2838.55±55.85a	1744.21±156.01b	611.51±145.11c	706.61±51.61c	1.76%	1.85%	1.86%	0.74%
(E,E)-2,4-Heptadienal(D)	C7H10O	1032.1	613.927	4368.19±119.23a	944.61±97.81b	119.51±15.41c	216.91±27.71c	2.71%	1.00%	0.36%	0.23%
Benzaldehyde(M)	C7H6O	974.3	504.42	1067.01±25.24a	1115.11±68.61a	378.71±88.11b	430.31±29.41b	0.66%	1.18%	1.15%	0.45%
Benzaldehyde(D)	C7H6O	974.8	505.399	439.25±2.57a	418.71±22.51a	127.81±8.11b	136.01±2.21b	0.27%	0.44%	0.39%	0.14%
(E)-2-Heptenal(M)	C7H12O	962.7	482.412	2437.66±33.25a	2022.31±42.81b	712.81±57.21d	994.11±40.71c	1.51%	2.14%	2.17%	1.04%
(E)-2-Heptenal(D)	C7H12O	960.8	478.989	5721.49±169.50a	1463.81±90.51b	225.71±23.51d	639.91±64.01c	3.55%	1.55%	0.69%	0.67%
(E,E)-2,4-Hexadienal(M)	C6H8O	921.2	411.261	708.41±29.51a	278.41±20.11b	167.31±21.21c	254.51±20.21b	0.44%	0.29%	0.51%	0.27%
(E,E)-2,4-Hexadienal(D)	C6H8O	919.5	408.621	540.33±18.57a	64.71±3.71b	70.71±2.41b	81.41±4.51b	0.34%	0.07%	0.22%	0.09%
(E)-2-Hexenal	C6H10O	844.4	309.913	30304.03±263.37a	7199.51±780.51c	1487.41±170.21d	20217.61±850.11b	18.81%	7.62%	4.53%	21.13%
Hexanal	C6H12O	790.7	255.698	4330.80±80.76c	8187.61±80.61a	6254.61±508.81b	6866.81±208.91b	2.69%	8.66%	19.05%	7.18%
(E)-2-Pentenal(M)	C5H8O	749.8	218.87	1307.64±168.13c	1785.11±103.71a	1438.21±66.81bc	1627.31±44.91ab	0.81%	1.89%	4.38%	1.70%
(E)-2-Pentenal(D)	C5H8O	750.5	219.478	13278.51±228.67a	5581.91±59.51b	1122.11±180.71d	4907.61±414.81c	8.24%	5.91%	3.42%	5.13%
3-Methylbutanal	C5H10O	654.3	156.409	198.21±16.31b	36.21±3.01c	18.71±0.51c	455.61±19.81a	0.12%	0.04%	0.06%	0.48%
Octanal	C8H16O	1011	574.742	420.85±10.31a	394.11±1.61b	341.31±18.41c	347.01±1.61c	0.26%	0.42%	1.04%	0.36%
2,4-Heptadienal	C7H10O	1014.6	581.257	1169.56±174.37a	476.61±143.31b	90.21±61.71c	120.21±15.01c	0.73%	0.50%	0.27%	0.13%
Pentanal	C5H10O	694.4	176.941	184.98±7.15c	210.31±8.91bc	732.21±19.71a	220.11±3.81b	0.11%	0.22%	2.23%	0.23%
Heptanal(M)	C7H14O	904.6	385.932	613.29±4.63c	822.41±17.91b	649.61±24.31c	941.51±17.41a	0.38%	0.87%	1.98%	0.98%
Heptanal(D)	C7H14O	901.8	381.712	765.65±26.36b	932.11±54.21a	169.41±15.51d	407.71±26.61c	0.48%	0.99%	0.52%	0.43%
(E,E) -2,4-Nonadienal	C9H14O	1188.1	997.469	934.54±9.10a	476.91±6.31b	146.61±10.51c	466.91±19.21b	0.58%	0.50%	0.45%	0.49%
Total content								53.85%	41.12%	52.62%	45.28%
**Alcohols**											
Ethanol(M)	C2H6O	477.3	92.825	4373.90±15.93c	6258.01±104.81a	1272.71±140.11d	5045.21±122.21b	2.71%	6.62%	3.88%	5.27%
Ethanol(D)	C2H6O	480	93.566	555.98±28.57c	1089.81±74.21b	30.61±2.41d	1651.31±29.11a	0.35%	1.15%	0.09%	1.73%
1-Octen-3-ol(M)	C8H16O	999.1	553.877	2255.76±108.54b	3352.31±141.81a	1102.21±175.91c	2511.31±35.71b	1.40%	3.55%	3.36%	2.62%
1-Octen-3-ol(D)	C8H16O	991.5	538.939	1839.04±43.44b	2437.71±102.01a	173.61±53.41d	565.51±19.21c	1.14%	2.58%	0.53%	0.59%
1-Octen-3-ol(T)	C8H16O	989.8	535.464	434.97±13.14a	322.31±11.71b	50.21±7.51d	141.61±5.51c	0.27%	0.34%	0.15%	0.15%
1-Hexanol(M)	C6H14O	881.3	353.796	992.98±5.26c	1645.51±85.61b	399.71±249.91d	2350.91±111.21a	0.62%	1.74%	1.22%	2.46%
1-Hexanol(D)	C6H14O	883.8	357.042	1037.89±56.21b	911.91±47.31b	79.21±3.21c	2923.31±212.71a	0.64%	0.96%	0.24%	3.05%
1-Pentanol(M)	C5H12O	769.8	236.316	269.35±92.21a	384.41±135.61a	204.81±31.21a	395.51±86.31a	0.17%	0.41%	0.62%	0.41%
1-Pentanol(D)	C5H12O	773.8	240.026	307.71±42.99a	268.21±103.41a	46.01±2.61b	190.51±35.71a	0.19%	0.28%	0.14%	0.20%
1-Butanol(M)	C4H10O	666.9	162.306	625.40±30.04b	375.31±7.91c	98.01±14.61d	738.01±10.91a	0.39%	0.40%	0.30%	0.77%
1-Butanol(D)	C4H10O	666.5	162.098	242.62±8.96b	73.71±9.01c	14.11±1.11d	492.11±13.21a	0.15%	0.08%	0.04%	0.51%
1-Propanol	C3H8O	568.9	121.591	350.63±14.89c	1653.11±69.61a	231.21±57.81c	604.71±86.51b	0.22%	1.75%	0.70%	0.63%
Total content								8.24%	19.86%	11.28%	18.40%

Table S1. *Cont.*

Volatile compounds	Molecular formula	Retention index	Rt [sec]	Peak Volume				Relative content			
				WD	WY	WG	WS	WD	WY	WG	WS
**Ketones**											
1-Octen-3-one(M)	C8H14O	983.2	522.027	1579.48±13.65a	372.01±41.31c	183.21±21.11d	1003.51±39.51b	0.98%	0.39%	0.56%	1.05%
1-Octen-3-one(D)	C8H14O	983.2	522.027	5758.75±188.52a	715.71±20.11b	110.81±36.91c	967.81±141.01b	3.57%	0.76%	0.34%	1.01%
2-Butanone(M)	C4H8O	585.3	127.612	894.44±12.04a	664.41±18.41b	642.41±30.11b	914.01±40.21a	0.56%	0.70%	1.96%	0.96%
2-Butanone(D)	C4H8O	588	128.651	1179.23±54.41a	1078.11±33.41b	129.61±13.81d	273.21±5.41c	0.73%	1.14%	0.39%	0.29%
Acetone	C3H6O	504.6	100.605	3290.34±94.48a	2472.21±102.91b	139.41±15.41c	3193.71±53.01a	2.04%	2.62%	0.42%	3.34%
Total content								7.88%	5.61%	3.67%	6.64%
**Esters**											
Ethyl hexanoate	C8H16O2	1002.4	559.659	292.63±20.13b	247.41±26.11b	84.81±3.41c	666.41±50.51a	0.18%	0.26%	0.26%	0.70%
Ethyl pentanoate	C7H14O2	893	368.996	368.78±10.16b	608.81±8.71a	248.41±18.51c	182.61±7.61d	0.23%	0.64%	0.76%	0.19%
Total content								0.41%	0.91%	1.02%	0.89%
**Furans**											
2-Pentylfuran	C9H14O	994.2	544.524	2592.39±252.54a	390.61±6.51c	1111.11±73.21b	1044.21±128.31b	1.61%	0.41%	3.38%	1.09%
Total content								1.61%	0.41%	3.38%	1.09%
**Others**											
1	*	1135.9	847.888	2541.26±489.67a	450.51±177.11b	197.81±7.61b	526.51±28.01b	1.58%	0.48%	0.60%	0.55%
2	*	1138.2	854.081	364.64±181.42a	50.31±0.91b	43.91±5.81b	41.91±3.01b	0.23%	0.05%	0.13%	0.04%
3	*	1105.6	771.601	1721.57±74.62b	5811.51±282.21a	497.21±254.91d	1049.61±57.81c	1.07%	6.15%	1.51%	1.10%
4	*	1106.8	774.425	211.71±20.42b	1263.01±165.31a	98.41±8.71b	102.81±5.81b	0.13%	1.34%	0.30%	0.11%
5	*	1095.9	748.776	682.68±49.32a	181.21±18.01c	67.41±26.51c	500.71±88.41b	0.42%	0.19%	0.21%	0.52%
6	*	1096.8	750.692	165.57±32.91a	163.21±16.91a	69.71±10.41b	113.61±11.71b	0.10%	0.17%	0.21%	0.12%
7	*	1090.9	737.203	590.18±53.52a	380.01±22.21b	110.21±26.61d	218.91±12.71c	0.37%	0.40%	0.34%	0.23%
8	*	1082.6	718.415	601.69±29.96a	548.81±13.31a	113.51±38.31c	242.61±26.21b	0.37%	0.58%	0.35%	0.25%
9	*	1055.9	661.087	916.78±42.77a	143.11±16.71c	103.21±8.81c	565.31±2.11b	0.57%	0.15%	0.31%	0.59%
10	*	1044.6	638.258	1044.73±7.93a	823.21±14.41b	263.71±43.41d	615.11±19.01c	0.65%	0.87%	0.80%	0.64%
11	*	1044.5	638.004	856.80±13.22a	222.61±3.11c	153.11±4.81d	245.21±13.11b	0.53%	0.24%	0.47%	0.26%
12	*	1022.9	596.495	325.37±34.43a	216.41±5.41b	129.71±27.41c	232.61±12.71b	0.20%	0.23%	0.40%	0.24%
13	*	1022.7	596.099	150.40±16.23a	130.51±5.71ab	131.11±4.81ab	110.81±1.71b	0.09%	0.14%	0.40%	0.12%
14	*	1007.8	569.158	4958.09±282.80a	625.91±49.11c	325.31±75.01c	1723.21±152.61b	3.08%	0.66%	0.99%	1.80%
15	*	994.9	545.991	2440.11±39.71a	774.51±30.21b	270.91±54.81d	619.91±62.91c	1.51%	0.82%	0.83%	0.65%
16	*	988.5	532.786	1276.26±73.37b	4272.41±214.01a	720.51±220.21c	980.11±41.91bc	0.79%	4.52%	2.19%	1.02%
17	*	951.3	461.871	321.80±3.29a	59.51±19.61c	26.61±3.01d	109.31±4.51b	0.20%	0.06%	0.08%	0.11%
18	*	989.2	534.254	728.30±11.31c	911.91±23.31b	1237.91±65.31a	606.61±26.01d	0.45%	0.96%	3.77%	0.63%
19	*	898.3	376.693	1944.24±5.31b	2583.41±38.31a	533.71±32.71d	647.31±70.51c	1.21%	2.73%	1.63%	0.68%
20	*	892.8	368.77	256.88±4.69a	179.51±2.21b	84.01±8.11c	255.41±9.41a	0.16%	0.19%	0.26%	0.27%
21	*	893.4	369.675	1116.75±11.30b	417.81±9.61c	98.41±5.01d	1216.41±2.21a	0.69%	0.44%	0.30%	1.27%
22	*	870.4	340.245	256.58±24.18a	71.01±39.71b	33.21±2.31b	311.51±63.91a	0.16%	0.08%	0.10%	0.33%
23	*	824.8	288.91	325.50±30.05a	31.31±5.91c	20.81±2.31c	93.31±11.51b	0.20%	0.03%	0.06%	0.10%
24	*	808.9	272.911	61.95±1.47b	153.41±10.91a	67.21±9.11b	60.51±1.61b	0.04%	0.16%	0.20%	0.06%
25	*	821.2	285.181	109.92±1.11a	89.31±2.11b	67.21±3.51c	116.11±5.31a	0.07%	0.09%	0.20%	0.12%
26	*	774.5	240.609	236.03±3.85c	417.81±50.81b	903.71±119.81a	393.71±72.61bc	0.15%	0.44%	2.75%	0.41%

Table S1. *Cont.*

Volatile compounds	Molecular formula	Retention index	Rt [sec]	Peak Volume				Relative content			
				WD	WY	WG	WS	WD	WY	WG	WS
27	*	728.7	201.839	147.03±2.39b	106.71±2.71c	172.11±3.81a	126.31±17.31bc	0.09%	0.11%	0.52%	0.13%
28	*	728.2	201.433	120.41±1.03a	19.71±3.01c	9.91±0.61d	52.01±3.51b	0.07%	0.02%	0.03%	0.05%
29	*	717.5	193.323	105.80±7.73a	111.41±4.21a	101.91±11.21a	77.71±11.11b	0.07%	0.12%	0.31%	0.08%
30	*	721.5	196.364	53.57±5.52a	14.91±2.21b	11.41±2.31b	15.31±2.31b	0.03%	0.02%	0.03%	0.02%
31	*	679.9	168.616	10111.51±16.73a	2526.11±78.71c	555.21±34.11d	7970.31±321.61b	6.27%	2.67%	1.69%	8.33%
32	*	617.6	140.367	242.19±6.45b	401.21±11.51a	164.01±3.21c	253.11±8.71b	0.15%	0.42%	0.50%	0.26%
33	*	544.4	113.138	158.14±7.22b	109.91±8.81c	41.41±0.51d	763.61±6.81a	0.10%	0.12%	0.13%	0.80%
34	*	944.7	450.25	485.73±4.17a	224.01±22.51c	111.61±16.11d	306.11±62.91b	0.30%	0.24%	0.34%	0.32%
35	*	527.5	107.625	56.02±1.14b	24.71±2.41c	11.21±1.01d	275.91±7.91a	0.03%	0.03%	0.03%	0.29%
36	*	1096.6	750.364	555.49±18.96a	576.41±35.01a	120.21±42.81b	187.31±28.21b	0.34%	0.61%	0.37%	0.20%
37	*	923	414.173	203.25±3.50b	114.51±5.71c	50.01±6.11d	293.31±6.81a	0.13%	0.12%	0.15%	0.31%
38	*	698.9	179.995	1462.10±74.93a	485.31±7.71c	445.31±22.11c	1019.61±56.91b	0.91%	0.51%	1.36%	1.07%
39	*	703.2	183.016	860.86±4.09b	1247.31±59.31a	221.01±28.41d	674.51±31.71c	0.53%	1.32%	0.67%	0.70%
40	*	646.8	152.957	3965.45±28.20a	2034.21±81.71b	264.31±27.41d	1091.51±31.81c	2.46%	2.15%	0.81%	1.14%
41	*	1044.6	638.194	1187.40±30.18a	1021.61±22.71b	472.31±41.61c	1221.01±26.31a	0.74%	1.08%	1.44%	1.28%
42	*	1044.5	638.03	1199.92±43.06a	349.01±16.01c	79.41±13.51d	483.31±59.31b	0.74%	0.37%	0.24%	0.51%
Total content								28.00%	32.10%	28.03%	27.70%
